# Associations of hair cortisol concentrations with paediatric appendicitis

**DOI:** 10.1038/s41598-021-94828-8

**Published:** 2021-07-27

**Authors:** Johanna Gudjonsdottir, Michaela Runnäs, Lars Hagander, Elvar Theodorsson, Martin Salö

**Affiliations:** 1 Department of Clinical Sciences, Pediatrics, Lund University, Lasarettsgatan 48, 221 85 Lund, Sweden; 2 Department of Surgery, Skåne University Hospital, Malmö, Sweden; 3 Department of Paediatric Surgery, Skåne University Hospital, Lund, Sweden; 4 Department of Biomedical and Clinical Science, Clinical Chemistry, Linköping University, Linköping, Sweden

**Keywords:** Paediatrics, Gastrointestinal diseases, Paediatric research

## Abstract

The pathogenesis of paediatric appendicitis is still an enigma. In recent years, it has become more evident that our inherent immunological responses affect the trajectory of the disease course. Long-term stress has an impact on our immune system; however, it is practically and ethically challenging to prospectively track blood measurements of cortisol-levels in asymptomatic children should an acute appendicitis episode develop. The aim of this case–control study was therefore to evaluate the effect of increased stress measured as historical imprints in hair (hair cortisol concentrations [HCC]), on the risk of developing appendicitis and complicated appendicitis. 51 children (aged < 15 years) with appendicitis (34 with complicated appendicitis), were compared to 86 healthy controls. HCC reflecting the activity of the HPA-axis 0–3 and 4–6 months prior to sampling was evaluated and compared between groups as well as between the two measurements of each study subject. In the univariate analysis with both cases and controls, an increase in HCC between the measurement timepoints was associated with a substantial increase in risk of appendicitis (OR 7.52 [95% CI 2.49–22.67], *p* = 0.001). This increased risk remained in the multivariate analysis after adjustment for age, sex and season (aOR OR 10.76 [95%CI 2.50–46.28], *p*  = 0.001). When comparing the cases of uncomplicated and complicated appendicitis through a multivariate analysis, adjusted for age and sex, the children with an increased HCC prior to appendicitis had a substantial and statistically significant increase in risk of complicated appendicitis (aOR 7.86 [95% CI 1.20–51.63], *p* = 0.03). Biological stress, measured as an increase in HCC, seems to be associated with an increased risk of paediatric appendicitis and a more complicated disease course.

## Introduction

Acute appendectomy is one of the most commonly performed surgeries globally^1,2^. Consensus regarding the aetiology behind the disease is, however, still lacking. The most established theory of an obstruction as a primary event^3,4^ has been questioned^5–7^. Instead, reports have indicated a role of a primary infectious event^8^, changes in the gut microbiome^9^, seasonal variations^10–12^, and disease occurrence in clusters^13^.

Another question is what drives the inflammation towards a complicated or an uncomplicated course of disease. Some children have a mild, sometimes self-limiting, appendicitis, whereas others follow a disease course that progresses towards perforation^14^. Clinical and epidemiological studies suggest that this discrepancy depends on immunology, where the uncomplicated disease is propelled by a T-helper (Th) cell 2-driven immune response, while the complicated disease trajectory is driven by a Th1- and Th17-driven immune response^15–17^. This raises the question, whether the disease course of appendicitis is dependent on genetic factors that creates a shift in our innate and adaptive immune systems^15,18^ or if the immunological shifts are dependent on other circumstances, such as pregnancy^19^.

Stress, both acute and chronic, is a well-known immune modulator^20^, and seems to be an important factor in the pathophysiology of a range of diseases^21–24^. Stress results in alterations in the activity of the hypothalamic–pituitary–adrenal (HPA)-axis, which in turn leads to increased secretion of the glucocorticoid cortisol. Cortisol is most commonly measured in saliva, serum, or urine, but it would be practically and ethically challenging to prospectively track blood measurements of cortisol-levels in asymptomatic children should an acute appendicitis episode develop. Furthermore, the assessments are greatly affected by the acute context (i.e. circadian rhythm and daily variation), making them poor reflectors of long-term cortisol secretions^25^. Interestingly, however, historical levels of cortisol are continuously incorporated into the hair strands on the scalp as they grow from the roots, and the measurement of hair cortisol concentrations (HCC) is now a well-established marker for long-term endogenous cortisol concentrations^25,26^.

Glucocorticoids seem to suppress the Th1-cellular immunity axis and create a shift towards Th2-mediated immune response, rather than general immunosuppression^27,28^. This shift is, for example, supported by the association of Addison’s disease with an increased Th1 activity^29^. Given this, one hypothesis could therefore be that children with high levels of HCC would have a lower risk of Th1-/Th17-associated complicated appendicitis, since increased cortisol secretion seems to shift the immune system towards a Th2-driven response.

Thus, the aim of this study was to investigate the levels of HCC prior to disease onset in children with appendicitis and to compare the HCC in children with complicated and uncomplicated appendicitis.

## Method

### Study design

Children (< 15 years of age) with appendicitis and healthy controls were enrolled prospectively in the study between 2017 and 2019. The study was performed at a tertiary centre of paediatric appendicitis with an uptake area of 350,000 inhabitants for primary surgical care. At inclusion, a hair sample was collected from the posterior vertex area, and a questionnaire was filled out with help from one of the authors. The questionnaire covered the numbers of viral or bacterial infections, surgeries, and serious life events during the past 6 months. Serious life events were defined as, for example, parental divorce, domestic abuse, or the passing of a close relative. The study was approved by the regional ethical committee (Regionala Etikprövningsnämnden, Lund, Sweden, DNR 2017/242) and by the hospital review board (Skåne University Hospital, Lund, Sweden). The study was performed in accordance with relevant guidelines and regulations. The included subjects all agreed to participate through parental informed consent.

### Inclusion and exclusion criteria

All children < 15 years of age treated for appendicitis, surgically or with antibiotics (in the case of appendicular abscess), were eligible for inclusion in the study. Healthy children with no prior history of appendicitis were eligible for inclusion as controls. The controls were recruited through personal contacts, from hospital staff, and by advertisement at the local food markets and a family gym. Neither the cases nor the controls were reimbursed for their participation. The exclusion criteria were hair shorter than 6 cm in the posterior vertex area, previous episode of appendicitis, immunosuppressive/anti-inflammatory medication, chronic severe illness, or unwillingness to participate. The children with appendicitis were enrolled during their in-hospital stay.

### Primary outcome, exposure and independent variables

The primary outcome was complicated or uncomplicated appendicitis. The diagnosis was confirmed perioperatively and through histopathological examinations, or by radiology in the case of a conservatively managed appendicular abscess. Phlegmonous appendicitis was considered as uncomplicated, whereas gangrenous or perforated appendicitis as well as appendicular abscess were considered as complicated appendicitis. Phlegmonous appendicitis was defined as a neutrophilic infiltration of the muscularis propria^5^. Gangrenous appendicitis was defined as a transmural inflammation on histology, with necrosis seen macroscopically as grey or black discoloration, and the absence of perforation criteria^30^. Perforation was defined as an inflamed appendix with the presence of a visual hole in the appendix, or with the intraoperative finding of pus or an appendicolith in the abdominal cavity^31^.

The primary exposure was HCC 6–4 and 3–0 months prior to hair sampling, as well as the percentage difference in HCC between months 6–4 and 3–0. We investigated hair cortisol concentrations of the 6 cm of hair closest to the scalp, reflecting the cortisol exposure approximately 6 months prior to inclusion. This time frame was chosen in order to evaluate biological stress during as long a time frame as possible without excluding too many children due to short hair, especially boys. Independent variables were age, gender, number of viral or bacterial infections, surgeries, serious illnesses, and serious life events 6 months prior to hair sampling.

### Extraction of cortisol from hair samples

The hair strands covering an area of approximately 0.5 cm^2^ of the posterior vertex area were cut as close to the scalp as possible, in accordance with the recommendations from the Society of Hair Testing^32^. The ends closest to the scalp were tied together using aluminium foil. The hair was stored in plastic tubes at room temperature for up to 24 months before the analyses. The hair strands were sectioned into two parts of 3 cm, measured from the scalp end, reflecting the exposure during the 0–3 and 4–6 months prior to sampling, since hair grows at a rate of approximately 1 cm per month. The rationale behind splitting the hair samples into two measuring points is that we wanted to assess the effects of increased or decreased biological stress as estimated by HCC prior to the appendicitis episode. The HCC were analysed in methanol extracts of pulverised hair using an in-house competitive radioimmunoassay (RIA) and expressed as pg/mg.

The hair samples were weighed on a Sartorius MC 210p microscale (Sartorius Lab Instruments GmbH & Co, Göttingen, Germany) in 2 mL QiaGen RB sample tubes (Germantown, MD, USA). A 5 mm steel ball was added to each tube, after which the tubes were frozen in liquid nitrogen for 2 min and minced rapidly at 30 Hz for 20 s in a Retch TissueLyser II (Retsch GmbH, Haan, Germany), leaving a fine hair powder. 1 mL pure ethanol (Chromasolv, Sigma-Aldrich) was added to each test tube, and the test tubes were secured on a horizontal stirrer (Edmund Bühler, type B1) at room temperature for at least 10 h. Then the tubes were centrifuged and 700 μL of the supernatant of each sample was moved to another sample tube for lyophilisation in SpeedVac Plus SC210A (ThermoFisher, Scientific Inc, Bothell, WA, USA) using an Edwards XDS 5 vacuum pump.

### Measuring cortisol in the methanol extracted hair samples

The lyophilised extracts of hair samples were dissolved in 150 μL 0.1 mol/L phosphate buffer, pH 7.4, containing 0.02% bovine serum albumin and 0.01% triton X-100, and cortisol concentrations were analysed as described by Morelius et al.^33^ Hair samples of 3–10 mg were required in order to maintain a repeatability coefficient of variation below 8% for the combination of hair extraction and measurement of cortisol by the RIA.

### Statistics

All statistical analyses were performed in IBM SPSS for Macintosh, version 25.0 (Armonk, NY: IBM Corp). Continuous non-normal distributed variables were reported as median (min–max), and differences between groups were assessed using the Mann–Whitney U-test. Dichotomous variables were presented as frequencies and percentages, and differences between groups were assessed using the Chi-squared test. The percentage difference in HCC between months 4–6 and 0–3 was calculated by dividing the value of HCC 4–6 months with the value of HCC 0–3 months. The values of HCC at month 0–3 and 4–6 were logarithmised before further analysis. The effect of HCC levels, at 0–3 months, 4–6 months, and the percentage difference in HCC, on (a) appendicitis and b) complicated appendicitis, were measured through univariate and multiple logistic regression and reported as odds ratios (OR) with 95% confidence intervals (95% CI). Independent variables included in the multiple regression analyses were age, sex, and any other significant (*p* < 0.05) variable from the univariate analyses.

## Results

**Figure 1:**
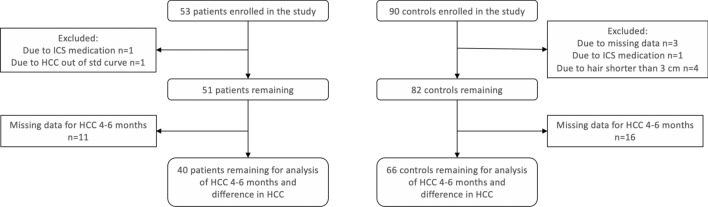
Flowchart over inclusion and exclusion of patients and controls.

53 patients with appendicitis and 90 controls were enrolled in the study. After exclusion due to missing data, ongoing corticosteroid medication, and HCC values outside the standard curve, a total of 51 cases and 82 controls remained for further analyses. Of these, 11 patients and 16 controls were further excluded from the analysis of HCC between 4 and 6 months because the hair was too short. Hence 40 patients and 66 controls remained for analysis of HCC 4–6 months and difference in HCC (Fig. 1). Among the 34 cases of complicated appendicitis, there were three gangrenous and 19 perforated cases of appendicitis, and 12 had an appendicular abscess. Seventeen children had uncomplicated (phlegmonous) appendicitis.

When including both cases and controls, children with high HCC were significantly younger, were more often affected by complicated appendicitis, and had been included less often during the winter period (Table 1). When only including cases of appendicitis, children with a high HCC were younger and were more often affected by complicated appendicitis (Table 2). There were no significant differences in any of the two analyses between children with high and low HCC regarding sex, viral or bacterial infections, surgeries, other medical conditions, or serious life events.

**Table 1:** Level of hair cortisol concentration in 51 patients with appendicitis and 82 healthy controls.

	Low HCC (n = 66)	High HCC (n = 67)	*p* value
**Age** (years)	9 (1–14)	5 (1–15)	0.001*
**Sex** (Male)	25 (38%)	35 (52%)	0.10**
**Viral infections** < 6 m	1 (0–6)	2 (0–8)	0.15*
**Bacterial infections** < 6 m	0 (0–1)	0 (0–2)	0.06*
**Surgery** < 6 m	1 (2%)	1 (1%)	0.99**
**Serious illness** < 6 m	4 (6%)	2 (3%)	0.39**
**Serious life events** < 6 m	2 (3%)	2 (3%)	0.99**
**Season**			0.038**
*Winter*	23 (35%)	10 (15%)	0.008**
*Spring*	6 (9%)	5 (7%)	0.73**
*Summer*	13 (20%)	14 (21%)	0.86**
*Autumn*	24 (36%)	38 (57%)	0.019**
**Appendicitis**	28 (42%)	23 (34%)	0.34**
*Complicated appendicitis*	15 (54%)	19 (83%)	0.029**

Values presented as median (min–max) or as absolute number (percentage of patients/controls); HCC: hair cortisol concentration; Low HCC < 22.96 pg/mg (median), high HCC ≥ 22.96 pg/mg; *Mann–Whitney U-test, **Chi-squared test.

**Table 2:** Level of hair cortisol concentration in 51 patients with appendicitis.

	Low HCC (n = 25)	High HCC (n = 26)	*p* value
**Age** (years)	10 (4–13)	5.5 (1–13)	0.03*
**Sex** (Male)	12 (48%)	14 (54%)	0.68**
**Viral infections** < 6 m	1 (0–4)	2 (0–5)	0.42*
**Bacterial infections** < 6 m	0 (0–1)	0 (0–2)	1.0*
**Surgery** < 6 m	0 (0%)	0 (0%)	–
**Serious illness** < 6 m	3 (12%)	2 (8%)	0.61**
**Serious life events** < 6 m	2 (8%)	2 (8%)	0.97**
**Season**			0.17**
*Winter*	5 (20%)	3 (12%)	0.41**
*Spring*	3 (12%)	6 (23%)	0.30**
*Summer*	6 (24%)	6 (23%)	0.94**
*Autumn*	11 (44%)	11 (42%)	0.90**
**Complicated appendicitis**	13 (52%)	21 (81%)	0.03**

Values presented as median (min–max) or as absolute number (percentage of patients/controls); HCC: hair cortisol concentration; Low HCC: < 21.03 pg/mg (median), high HCC ≥ 21.03 pg/mg (median; *Mann–Whitney U-test, **Chi-squared test.

**Figure 2:**
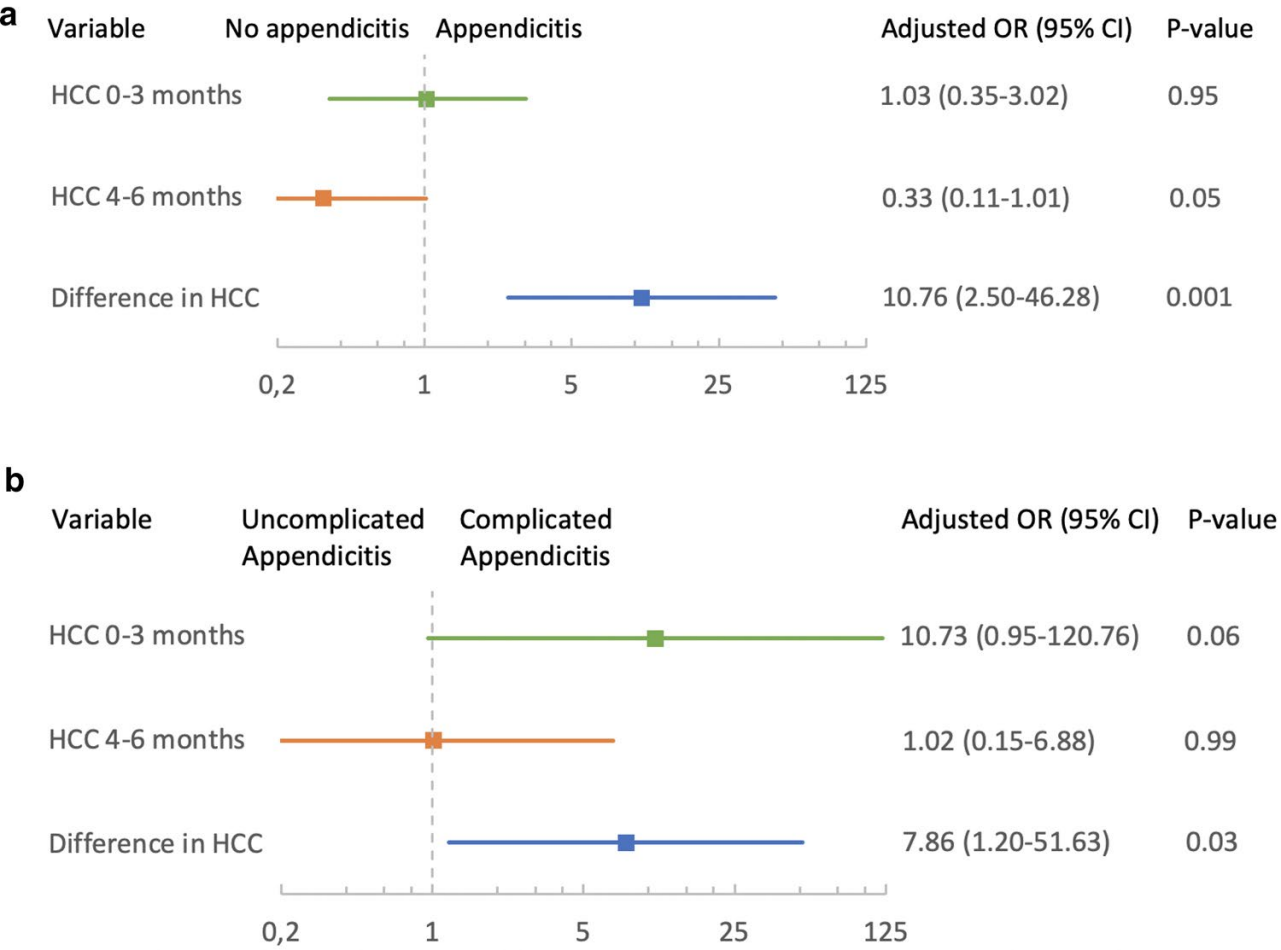
(**a**) Adjusted odds ratio for appendicitis for different timepoints of hair cortisol concentrations in 51 children with appendicitis and 82 controls. n = 106 (66 controls and 40 cases) for HCC 4–6 months and difference in HCC. Adjusted for age, sex and season. (**b**) Adjusted odds ratio for appendicitis for different timepoints of hair cortisol concentrations in 51 children with appendicitis. n = 40 for HCC 4–6 months and difference in HCC. Adjusted for age and sex.

In the univariate analysis with cases and controls, high values of HCC 0–3 and 4–6 months prior to appendicitis were not associated with an increased risk of appendicitis (Table 3) and did not affect the risk of appendicitis significantly in the multivariate analysis (Fig. 2a). However, an increase in HCC between the measurement timepoints was associated with a significant increase in risk of appendicitis (OR 7.52 [95% CI 2.49–22.67], *p* = 0.001) (Table 3). This risk increase remained in the multivariate analysis after adjustment for age, sex and season (adjusted (a) OR 10.76 [95%CI 2.50–46.28], *p* = 0.001) (Fig. 2a).

**Table 3:** Unadjusted independent variables of appendicitis in 51 children with appendicitis and 82 healthy controls.

	Appendicitis (n = 51)	Controls (n = 82)	OR (95% CI)	*p* value
**Age** (years)	9 (1–13)	6 (1–15)	1.11 (1.02–1.22)	**0.021**
**Sex** (Male)	26 (51%)	38 (41%)	0.68 (0.34–1.37)	0.284
**Viral infections** < 6 m	2 (0–5)	2 (0–8)	0.94 (0.74–1.19)	0.600
**Bacterial infections** < 6 m	0 (0–2)	0 (0–1)	1.16 (0.30–4.48)	0.825
**Surgery** < 6 m	0 (0%)	2 (2%)	–	–
**Serious illness** < 6 m	5^a^ (10%)	1^b^ (1%)	8.8 (0.998–77.67)	0.050
**Serious life events** < 6 m	4^c^ (8%)	0 (0%)	–	–
**Season**				**0.021**
*Winter*	8 (16%)	25 (30%)	0.58 (0.23–1.51)	0.264
*Spring*	9 (18%)	2 (2%)	8.18 (1.62–41.26)	**0.011**
*Summer*	12 (24%)	15 (18%)	1.45 (0.58–3.65)	0.425
*Autumn*	22 (43%)	40 (49%)	1	
**HCC 0–3 months** (*pg/mg)*	21.3 (6.7–373.7)	24.1 (6.0–606.7)	0.89 (0.37–2.15)	0.80
**HCC 4–6 months** (*pg/mg)*	13.7 (4.1–1524.5)	25.1 (5.8–979.12)	0.46 (0.19–1.10)	0.081
**Difference in HCC** (%)	123 (16–441)	100 (15–182)	7.52 (2.49–22.67)	**0.001**

Values presented as median (min–max) or as absolute number (percentage of patients).

M: male, F: female. n = 106 (66 controls, 40 cases) for HCC 4–6 months and difference in HCC.

a) 2 fractures, 1 thyrotoxicosis, 1 severe autism, 1 severe ADHD; b) urinary tract disorder with long-term catheter; c) 1 with severely depressed father, 2 with domestic abuse, 1 with loss of close relative.

When only including cases of appendicitis, univariate logistic regression showed that high HCC during the 0–3 months before the appendicitis episode was associated with a significantly increased risk of complicated appendicitis (OR 18.04 [95% 2.04–159.64], *p* = 0.01) (Table 4). This risk was no longer significant when adjusting for age and sex in the multivariate analysis (aOR 10.73 [95%CI 0.95–120.76], *p* = 0.055) (Fig. 2b). No increased risk for complicated appendicitis was found for HCC at 4–6 months, neither in the univariate nor the multivariate analysis (Fig. 2b). In the univariate analysis, an increase in HCC was not significantly associated with an increased risk of complicated appendicitis (Table 4). In the multivariate analysis, however, an increase in HCC between the two measurement timepoints was associated with a significant increase in risk for complicated appendicitis (aOR 7.86 [95% CI 1.20–51.63], *p* = 0.03) (Fig. 2b).

**Table 4:** Unadjusted independent variables of complicated disease in 51 children with appendicitis.

	Complicated (n = 27)	Uncomplicated (n = 14)	OR (95% CI)	*p* value
**Age** (years)	8.5 (1–13)	11 (4–13)	0.76 (0.62–0.94)	**0.01**
**Sex** (Male)	17/17	9/8	1.13 (0.35–3.61)	0.84
**Viral infections** < 6 m	2 (0–5)	1 (0–5)	1.31 (0.8–2.13)	0.29
**Bacterial infections** < 6 m	0 (0–2)	0 (0–0)	–	–
**Surgery** < 6 m	0	0	–	–
**Serious illness** < 6 m	2 (6%)	3 (18%)	0.29 (0.04–1.94)	0.20
**Serious life events** < 6 m	2 (6%)	2 (12%)	0.47 (0.06–3.66)	0.47
**Season**				0.22
*Winter*	3 (9%)	5 (29%)	0.28 (0.05–1.52)	0.14
*Spring*	8 (24%)	1 (6%)	3.73 (0.39–35.93)	0.25
*Summer*	8 (24%)	4 (24%)	0.93 (0.21–4.18)	0.93
*Autumn*	15 (44%)	7 (41%)	1	
**HCC 0–3 months** (*pg/mg)*	32.4 (8.4–373.7)	14.3 (6.7–35.3)	18.04 (2.04–159.64)	**0.01**
**HCC 4–6 months** (*pg/mg)*	14.20 (4.1–1524.5)	9.80 (5.1–80.1)	2.53 (0.53–11.99)	0.24
**Difference in HCC** (%)	137 (16–4.41)	129 (38–205)	2.91 (0.82–10.34)	0.10

n = 40 for HCC 4–6 months and difference in HCC.

## Discussion

This is the first ever study evaluating the effects of increased stress, measured as HCC, on the risk of appendicitis. We found that an increase in HCC is associated with a significant increase in the risk of appendicitis and complicated appendicitis in children. These findings indicate that biological stress, as measured by HCC, might be a new clinically significant risk factor for appendicitis and complicated appendicitis in children.

Appendicitis has long been considered a uniform disease in which the inflammation progresses until perforation if left untreated. In recent years, this view of the natural history of appendicitis has been questioned and it has become increasingly evident that instead appendicitis consists of two different disease entities: the sometimes self-limiting uncomplicated (phlegmonous) form and the complicated (gangrenous appendicitis, perforated appendicitis and appendicular abscess) form^14^. It seems that a person’s immune system is involved in driving the inflammation in one or the other direction, where complicated appendicitis is associated with a Th1- and Th17-driven immune response; this is supported by the finding of increased levels of Th17-associated cytokines in patients with gangrenous appendicitis^34^. This is further supported by the increased risk of the Th1-associated disease Morbus Crohn’s in patients who have undergone an appendectomy^35^; however, this increased risk seems to be transient and might be the result of diagnostic bias^36^. On the other hand, associations have been found between uncomplicated appendicitis and pregnancy^19^ and IgE-mediated allergy^15^, both Th2-associated conditions. An inverse relationship has also been found between appendicitis and the Th2-associated disease ulcerative colitis^37^. Since stress mediates a shift towards a Th2-dominated immunity^27^, we hypothesised that children with high levels of HCC would be protected against complicated appendicitis. When comparing the children with appendicitis, we found the opposite relationship, where an increase in HCC was significantly associated with complicated appendicitis.

This study may seem to raise more questions than it answers, and we can only speculate on which mechanisms underly the association of elevated levels of HCC and appendicitis in children. Previous studies have reported a stress-induced intestinal barrier dysfunction^38,39^, and one could therefore hypothesise that this also might affect the appendix and subsequently increase the risk of bacterial translocation. One could also speculate over the seasonal variation of both HCC and appendicitis. It is possible that the seasonal variance in the incidence and/or severity of appendicitis to some extent is affected by the variations in HCC. The same reasoning can be applied to the impact of age on the risk of complicated appendicitis. In our cohort, the children with low levels of HCC were significantly older than the children with high HCC, and the young children were affected by complicated appendicitis to a greater extent.

The results of this study have to be interpreted in the light of its limitations. First of all, the inclusion of cases on the ward was carried out by three of the authors, who were not always available. Some patients were therefor discharged before being asked about participation in the study. We have a large proportion of complicated appendicitis in our cohort (67%), compared to previous figures reported from our centre (42%)^15^, indicating a selection bias caused by the rapid postoperative discharge of patients with uncomplicated appendicitis. The self-reported nature of the data on comorbidities is another weakness. We have not included weight in our analysis, another factor that has previously been reported to be associated with HCC^40^. To our knowledge, obesity does not affect the risk of appendicitis but perhaps influences the severity^41^. Exposing hair to water and shampoo have been shown to decrease HCC in the more distal parts of the hairs^42^ – an effect that was not controlled for in the present study. A study to be published from our laboratory found wash-out effects of HCC to be small, even for those of the 48 women followed for 24 months who reported that they washed their hair every day. Chances in HCC were more likely to be related to stressors than to other cause (Faresjö, A., personal communication).

Finally, a power calculation was not performed before the study since no prior studies could provide an estimate of expected changes in HCC.

The strength of the study is its originality. Translational studies on the pathogenesis underlying appendicitis are lacking, which would help improve our knowledge of the disease^43^. The cases in this study were included in the paediatric surgery ward during the patients’ postoperative stay after appendectomy, or during their treatment for appendicular abscess. This constitutes a great stressor, but since the hair strands originate from hair follicles located 3–5 mm beneath the scalp surface^44^, the previous couple of days’ growth of hair lie just beneath the scalp, and the stress related to the appendicitis itself should not have interfered with the HCC in the collected samples. We collected data on viral and bacterial infections, surgeries, serious illnesses and serious life events 6 months prior to inclusion to evaluate the effect of these events on HCC. Since there are no known associations of either of these factors and appendicitis, and there was no difference in these parameters between different outcomes or exposure levels, they were not considered to be confounding factors.

## Conclusion

For the first time HCC has been measured in children with appendicitis, to evaluate the associations of increased biological stress and the risk of appendicitis and complicated appendicitis. Our results indicate that increased levels of HCC in the most recent months before hair sampling are substantially and statistically significant and are associated with an increased risk of developing appendicitis and a complicated course of disease. We believe that this increase in risk is somehow caused by the immunomodulating effects of an increased long-term activity in the HPA-axis. Further studies on the effects of HCC and biological stress on the risk of appendicitis would add important information on this newly discovered association and might help shed light on the physiological mechanisms underlying it.
